# Emerging Roles of N6-Methyladenosine (m6A) Epitranscriptomics in Toxicology

**DOI:** 10.1093/toxsci/kfab021

**Published:** 2021-02-22

**Authors:** Emir Malovic, Alyssa Ealy, Arthi Kanthasamy, Anumantha G Kanthasamy

**Affiliations:** Department of Biomedical Sciences, Parkinson’s Disorder Research Program, Iowa State University, Ames, Iowa 50011

**Keywords:** epitranscriptomics, N6-methyladenosine (m6A) methylation, writer, eraser, reader, protein modulator

## Abstract

Epitranscriptomics, the study of chemically modified RNAs, is a burgeoning field being explored in a variety of scientific disciplines. Of the currently known epitranscriptomic modifications, N6-methyladenosine (m6A) methylation is the most abundant. The m6A modification is predominantly regulated by 3 tiers of protein modulators classified as writers, erasers, and readers. Depending upon cellular needs, these proteins function to deposit, remove, or read the methyl modifications on cognate mRNAs. Many environmental chemicals including heavy metals, pesticides, and other toxic pollutants, are all known to perturb transcription and translation machinery to exert their toxic responses. As such, we herein review how the m6A modification may be affected under different toxicological paradigms. Furthermore, we discuss how toxicants can affect the 3 tiers of regulation directly, and how these effects influence the m6A-modified mRNAs. Lastly, we highlight the disparities between published findings and theories, especially those concerning the m6A reader tier of regulation. In the far-reaching field of toxicology, m6A epitranscriptomics provides another enticing avenue to explore new mechanisms and therapies for a diverse range of environmentally linked disorders and diseases.

The dynamic and reversible N6-methyladenosine (m6A) methylation is the most abundant internal modification of messenger RNAs (mRNAs) and noncoding RNAs, including micro RNAs, transfer RNAs, ribosomal RNAs, circular RNAs, small nucleolar RNAs, and small nuclear RNAs, as well as several long noncoding RNAs. This post-transcriptional RNA modification is widely conserved among most eukaryotes, such as plants, insects, yeast and mammals, and is also found within some viruses (Dominissini *et al.*, 2012; Fu *et al.*, 2014). The m6A modification accounts for 0.1%–0.4% of all adenosines in global cellular RNAs, with about 3–5 m6A sites per mRNA, and represents approximately 50% of all methylated ribonucleotides (Zhang *et al.*, 2019a). Despite the discovery of m6A and its observed prevalence among mRNA modifications in the 1970s (Desrosiers *et al.*, 1975; Wei *et al.*, 1975), the lack of proper methodology made it difficult to investigate its putative roles in post-transcriptional mRNA regulation (Dominissini *et al.*, 2012). In 1978, Sommer, Lavi, and Darnell, Jr. (Sommer *et al.*, 1978) would be the first to observe the decreased half-life of m6A mRNA; however, its only in the past few decades that the 3 tiers of regulation centering on the reversibility of this post-transcriptional methylation would be discovered. Bokar *et al.* (1997) revealed the “writer” tier of m6A regulation by characterizing the now known METTL3 (methyltransferase-like 3) enzyme that localizes in the nuclear speckles, isolated domains enriched with pre-mRNA processing machinery. The “eraser” tier of regulation shows that this mRNA methylation is reversible by the identification of 2 principal demethylases, FTO (fat mass and obesity-associated alpha-ketoglutarate-dependent dioxygenase) (Jia *et al.*, 2011) and ALKBH5 (ALKB homolog 5 RNA demethylase) (Zheng *et al.*, 2013), although other evidence suggests FTO is more specific to m6Am (N6,2′-O-dimethyladenosine) (Mauer *et al.*, 2017, 2019; Zaccara *et al.*, 2019), a 5′ UTR terminal modification at the mRNA cap. Finally, the “reader” tier was discovered as a regulatory control mechanism of m6A using methylated RNA baits in conjunction with affinity chromatography and mass spectrometry, which revealed 2 proteins exclusively bound to methylated RNAs, YTHDF2 and YTHDF3 (YT521-B homology domain family) (Dominissini *et al.*, 2012). Further investigations on the functions of these readers, which would also include YTHDF1 (Wang *et al.*, 2015), revealed that the methylation of adenosine in DRACH (D = G > A>U, R = G > A, H = U > A>C) consensus sequences primarily destabilized the mRNAs (Dominissini *et al.*, 2013; Linder *et al.*, 2015), decreasing their overall half-life in ribosomal translation pools (Figure 1) (Du *et al.*, 2016; Fu *et al.*, 2014; Wang *et al.*, 2014). These DRACH sequences can be found throughout mRNA sequences. The m6A modifications are found within the coding sequence of long exons, but mostly enriched near the stop codons and the 3′ UTR region. Although 5′ UTR enrichment is also observable, this signature is primarily from the m6Am modification (Fu *et al.*, 2014; Meyer *et al.*, 2015). This complex ensemble of m6A mRNA methylation suggests that this mechanism may be vital to a plethora of cellular processes, and such processes like proliferation and viral replication have already been extensively observed in cancer and viral biology, respectively (Fu *et al.*, 2014). By drawing on experimental models relevant to the toxicological sciences, we review these three tiers of m6A regulation to highlight their putative importance across a broad range of cellular processes, including oxidative homeostasis, apoptosis, and inflammation. As we will discuss herein, we think m6A epitranscriptomics is an enticing and burgeoning field of RNA biology that possesses many targets of regulation that could be exploited for understanding molecular mechanisms of toxicopathogenesis, as well as the development of novel therapeutic strategies for the myriad of environmentally linked chronic diseases.

**Figure 1.**
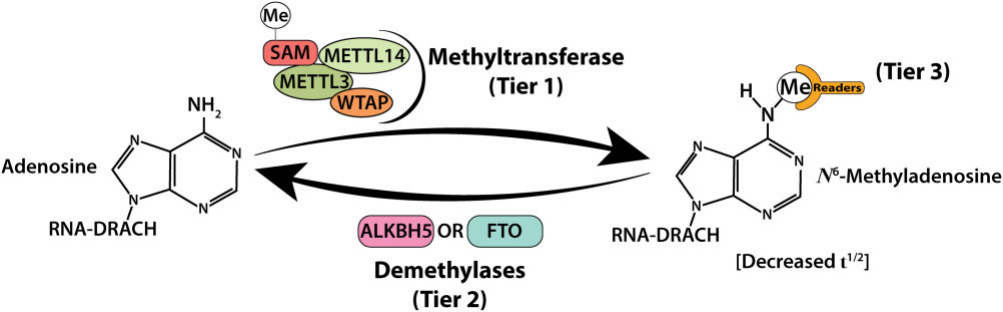
Chemical modification of adenosine. Adenosine bases in DRACH consensus sequences are co-transcriptionally modified into m6A bases by the methyltransferase complex (tier1) and can be demethylated by either ALKBH5 or FTO (tier 2). Readers (tier 3) bind to the methyl group and promote events such as translation or decay. Note: m6A mRNAs have decreased half-lives as compared with nonmethylated mRNAs, and thereby, m6A mRNA modification influences protein levels.

## PRINCIPAL WRITERS FOR M6A AND THEIR POTENTIAL ROLES IN TOXICOLOGY

The principal methylation writers, METTL3 and methyltransferase-like 14 (METTL14), form a complex with Wilm’s tumor 1-associating protein (WTAP) to deposit methyl groups on mRNAs (Langley *et al.*, 2017). This m6A methylation occurs co-transcriptionally when the writer complex binds to the DRACH consensus sequences. The heterodimer of METTL3 and METTL14 creates a pocket where the RNA and S-adenosylmethionine (SAM) are brought together. METTL3 catalyzes the methyl group deposition after SAM is positioned and stabilized by 11 residues through hydrogen bonding. METTL14 acts as the primary RNA scaffold by forming a positively charged groove with METTL3 consisting of mostly arginine residues. Mutation of METTL14’s Arg245, Arg255, Arg298, or Lys297 residues results in significant impairment of methyltransferase activity (Wang *et al.*, 2017), whereas mutation of METTL3’s residues Asp377 or Asp395 results in an undetectable binding activity of SAM (Wang *et al.*, 2016, 2017). Two CCCH zinc-binding motifs in METTL3 are also required for its proper methyltransferase activity (Wang *et al.*, 2016). Moreover, knockdown of METTL14 dramatically decreased RNA methylation, whereas the reintroduction of METTL14 sufficiently restored RNA methylation within the cell (Wang *et al.*, 2016). Thus, the RNA-binding interactions of METTL14 are required for the proper catalytic function of METTL3. Importantly, WTAP is responsible for proper substrate recruitment and METTL3/METTL14 localization to nuclear speckles, as this process, along with m6A RNA methylation, can be disrupted by knockdown of WTAP (Ping *et al.*, 2014).

The m6A epitranscriptomic levels and distribution can change depending on the cellular context and needs. Exposure to toxicants, such as the carcinogenic metal arsenite and particulate matter (PM_2.5_), as well as the endocrine disruptors bisphenol A (BPA) and vinclozolin, decreases global m6A methylation in a dose-dependent manner in the A549 adenocarcinoma cell line as reported by Cayir *et al.* (2019). They further corroborated their findings by showing that human lung adenocarcinoma and squamous cell carcinoma tissues had decreased protein levels of METTL3 with no significant changes in other m6A-related proteins. Such evidence would suggest the m6A writer levels are positively correlated with global m6A methylation levels; however, their gene expression analysis revealed increased METTL3 and WTAP with no changes in METTL14 when comparing humans exposed to high (6.0–119.0 μg/m^3^) versus low (7.6–55.1 μg/m^3^) levels of PM_2.5_. Furthermore, the m6A erasers ALKBH5 and FTO in high-exposure individuals were significantly upregulated, suggesting that reductions in global m6A methylation may also involve increased eraser levels (Cayir *et al.*, 2019). Indeed, zebrafish exposed to triclosan had decreased global m6A methylation resulting from significant changes in FTO levels, with no significant changes of METTL3 protein (Sun *et al.*, 2020). Despite such exceptions, m6A writer levels could be useful as preliminary indicators of global m6A methylation in specific model systems, including human cell lines (Chen *et al.,* 2019; Wang *et al.*, 2019b; Feng *et al.*, 2018; Ding *et al.*, 2020). Chen *et al.* (2019) observed *in vitro* that high arsenite doses decrease global m6A methylation by decreasing m6A writers while upregulating m6A erasers. Conversely, low arsenite doses increase global m6A methylation by increasing m6A writers while downregulating m6A erasers. In the *in vitro* case of using lipopolysaccharide (LPS) to experimentally induce inflammation, time-dependent treatment increases both METTL3 and global m6A methylation (Feng *et al.*, 2018; Wang *et al.*, 2019b). Lastly, reductions in global m6A methylation were achieved in the model cell lines HCC827 and H661 using the histone deacetylase inhibitor chidamide (Ding *et al.*, 2020). Western blot analyses of both cell models showed concomitant decreases in WTAP and METTL3 levels, but no significant changes in METTL14, nor the m6A eraser FTO. These studies collectively demonstrate that changes in the protein levels of METTL3, METTL14, or WTAP alone are sufficient to disrupt methyltransferase activity, especially in the event of their downregulation.

Many environmental stressors can generate inflammation and oxidative stress. LPS is a potent inducer of both (Qin *et al.*, 2013), and LPS treatment of MC3T3-E1 preosteoblasts in osteogenic medium reduces the levels of METTL3, suggesting that METTL3 has a protective effect in this particular cellular context (Zhang *et al.*, 2019b). Supporting this, METTL3 overexpression inhibits the LPS-induced inflammatory response of macrophages by interfering with the phosphorylation and nuclear translocation of NFκB in macrophages, thereby preventing the upregulation of the proinflammatory cytokines IL-6 and TNF-α (Wang *et al.*, 2019b). Some chemotherapeutic drugs induce oxidative stress, like colistin in the nephrotoxicity model using mouse renal tubular epithelial cells, which is marked by decreased SOD, CAT, and GSH-PX, as well as enhanced apoptosis as measured by activation of caspase-3 and caspase-9, cytochrome C release, and DNA fragmentation. Overexpressing METTL3 prevents colistin-induced oxidative stress and apoptosis as revealed by unaffected levels of SOD, CAT, GSH-PX, caspase-3 and -9, cytochrome C release, and DNA fragmentation (Wang *et al.*, 2019a). Colistin treatment also increases KEAP1 levels and decreases NRF2 and HO-1. NRF2 increases the transcription of antioxidant genes such as thioredoxin reductase 1 and peroxiredoxin 1 (Taguchi *et al.*, 2011). Interestingly, METTL3 regulates KEAP1 activation by binding to DGCR8 (DiGeorge Syndrome Critical Region 8) to promote DGCR8’s recognition of miR-873-5p. DGCR8 then recruits the RNase DROSHA, leading to maturation of miR-873-5p, ultimately leading to binding of miR-873-5p to KEAP1 and ensuing KEAP1 inactivation (Wang *et al.*, 2016). The ability of METTL3 to control the KEAP1-NRF2 pathway highly suggests that both METTL3 and its methyltransferase activity are intimately associated with hypoxic and oxidative stress responses. However, such responses can be concentration-dependent as in the case of arsenite. Low concentrations of arsenite increase the expression of METTL3 and METTL14; however, high-level exposure to arsenite decreases METTL3, WTAP, and METTL14 levels (Chen *et al.*, 2019; Xiong *et al.*, 2019; Zhao *et al.*, 2020). Furthermore, the cells exposed to higher concentrations of arsenite display increased oxidative stress with decreased cell viability and had higher levels of m6A demethylases (Chen *et al.*, 2019). In sum, m6A writer levels could provide an initial path in studies investigating m6A epitranscriptomics, but caution should be taken as certain paradigms have shown global m6A methylation changes that were dependent upon the principal m6A erasers.

## PRINCIPAL ERASERS FOR M6A AND THEIR POTENTIAL ROLES IN TOXICOLOGY

Initial observations of m6A cytoplasmic clearance through radiolabeling experiments suggested that m6A mRNAs were a class of rapidly degradable RNAs rather than being targets of demethylases (Sommer *et al.*, 1978). We now know this initial hypothesis to be true, in that demethylases are not responsible for the rapid clearance of m6A mRNAs in the cytoplasm (Du *et al.*, 2016; Park *et al.*, 2019), but 2 principal m6A demethylases do exist, FTO (Jia *et al.*, 2011) and ALKBH5 (Zheng *et al.*, 2013). Both demethylases belong to the same ALKB family of iron (Fe^2+^)- and α-ketoglutarate-dependent dioxygenases. In the case of FTO, mutation of Arg316, which is required for the binding stabilization of α-ketoglutarate, resulted in an 80% loss of m6A demethylation. Double mutation of the α-ketoglutarate-binding sites Arg316 and Arg322, or the Fe^2+^-binding sites His231 and Asp233, would result in a 100% loss of demethylation (Jia *et al.*, 2011). For ALKBH5, mutating one of the Fe^2+^-binding sites, His204 or His266, results in a 100% loss or approximately 80% loss of m6A demethylation activity, respectively (Zheng *et al.*, 2013). Though these principal loss-of-function studies highlight the specificity of FTO and ALKBH5 to m6A, other studies suggest FTO’s demethylation specificity to m6A is low (Garcia-Campos *et al.*, 2019; Mauer *et al.*, 2017). Specifically, FTO’s *k*_cat_ (turnover number) is approximately 20-fold greater for the 5′ UTR mRNA modification m6Am compared with m6A, even though FTO’s *k*_cat_ (0.296 min^−1^; Jia *et al.*, 2011) for m6A is comparable to that of ALKBH5 (0.169 min^−1^; Zheng *et al.*, 2013) (Mauer *et al.*, 2017). Additionally, because FTO is primarily localized in the nucleus, its function may be predominantly nuclear, as in the observed demethylation of small nuclear RNAs that regulate mRNA splicing (Mauer *et al.*, 2019). Despite this disparity in identifying FTO’s natural substrate, other researchers have continued to observe *in vivo* impacts of FTO on its cognate mRNAs (Li *et al.*, 2017b), suggesting that even if its specificity was low or that m6A was not its natural substrate, FTO can still demethylate said mRNAs to alter the physiological state of cells.

FTO’s and ALKBH5’s enzymatic activities depend on both Fe^2+^ and α-ketoglutarate levels (Gerken *et al.*, 2007; Zheng *et al.*, 2013) and have been implicated in numerous cellular processes like energy homeostasis, DNA repair, dopaminergic signaling, and so forth (Frayling *et al.*, 2007; Hess *et al.*, 2013; Li *et al.*, 2017b; Mishina and He, 2006; Zaccara *et al.*, 2019; Zheng *et al.*, 2013), making them enticing targets from a toxicological perspective. FTO’s expression is highest in the brain, specifically the hypothalamus (Gerken *et al.*, 2007), which also explains its widespread association with obesity and type-2 diabetes (Frayling *et al.*, 2007). Because of its significant expression in the brain and its iron dependency, FTO may be a desirable target in the aging brain. Iron levels gradually increase in the aging human basal ganglia, with the substantia nigra being a major site of accumulation (Zecca *et al.*, 2004; Zucca *et al.*, 2017). Excessive free iron can promote oxidative stress through the generation of hydroxyl radicals (Zecca *et al.*, 2004). Knowing how this age-related change affects the activity of FTO and its cognate mRNA targets would yield vital insights into the epitranscriptomics of neurodegenerative disorders like Parkinson’s disease (PD). The first genome wide association study assessing 1647 sporadic PD patients and 1372 controls of Chinese origin found no significant associations between the writer, eraser, or reader genes discussed herein with sporadic PD (Qin *et al.*, 2020). Despite this genetic analysis, several preliminary findings have shown that arsenic treatment can decrease both FTO and tyrosine hydroxylase (TH) levels in mice, whereas *in vitro* overexpression of FTO can prevent the arsenic-induced decrease of TH in the PC-12 cell line (Bai *et al.*, 2018). Interestingly, rats treated with 6-hydroxydopamine (6-OHDA) develop elevated levels of FTO in the midbrain, as do 6-OHDA-treated PC-12 cells (Chen *et al.*, 2019d); 6-OHDA is an established Parkinsonian neurotoxicant that induces dopaminergic neuronal death marked by dramatically decreased TH levels (Panicker *et al.*, 2015). Based on these examples, it is imperative to thoroughly investigate the correlation between FTO and TH levels in the context of PD and other Parkinsonian disorders as it could provide a new avenue of therapy. Our laboratory is presently studying the effect of m6A metabolism in a metal-induced Parkinsonism.

ALKBH5’s expression is comparatively greater than FTO’s in most tissues, whereas its greatest expression occurs in the testes (Gerken *et al.*, 2007; Zheng *et al.*, 2013). Indeed, ALKBH5-deficient mice have smaller testes with defects in spermatogenesis and concomitant apoptosis (Zheng *et al.*, 2013). Because ALKBH5’s expression is more widespread and its substrate specificity has not been contested, it may serve as an easier investigative target in diverse toxicological models (Cayir *et al.*, 2019). In fact, ALKBH5 has been observed as a gene target of HIF1α during hypoxia (Thalhammer *et al.*, 2011). Hypoxic cells exhibit less global m6A methylation, which could be attributed to significantly increased ALKBH5 (Chao *et al.*, 2020; Zhang *et al.*, 2016). In general, a decline in global m6A methylation suggests increased expression of ALKBH5, as reported in *in vitro* toxicological models of BPA, vinclozolin, particulate matter, and arsenic (Cayir *et al.*, 2019), of which arsenic can be a potent inducer of hypoxia (Al Taleb *et al.*, 2016; Liu *et al.*, 2020; Wang *et al.*, 2012). As noted above, both ALKBH5 and FTO require Fe^2+^ as a cofactor for catalysis, but they also require molecular oxygen (Aik *et al.*, 2014). Arsenic (Zhao *et al.*, 2019), heavy metals like iron and manganese (Carocci *et al.*, 2018; Sarkar *et al.*, 2018), and even pesticides can generate oxidative stress (Sarkar *et al.*, 2017). Further investigations are warranted into how such shifts in the oxygen balance affect the functionality of these m6A demethylases and their cognate m6A mRNAs.

## PRINCIPAL READERS FOR M6A AND THEIR POTENTIAL ROLES IN TOXICOLOGY

Over the last decade, numerous m6A-binding proteins have been discovered (eg, eIF3; Meyer *et al.*, 2015, IGF2BP; Huang *et al.*, 2018, HNRNPA2B1; Alarcon *et al.*, 2015, YTHDC2; Kretschmer *et al.*, 2018). Of these RNA-binding proteins, we only review the YTHDF (YT521-B homology domain family) paralogs in the context of their putative functions, potential redundancy, and association to toxicology. The YTHDF paralogs include YTHDF1, YTHDF2, and YTHDF3, which are localized to the cytoplasm. Investigations into the function of YTHDF2 revealed that it functions primarily to promote the degradation of m6A mRNAs (Wang *et al.*, 2014). YTHDF2 executes this by primarily reading polymethylated m6A mRNAs (Ries *et al.*, 2019) and inducing deadenylation by recruiting the CCR4-NOT deadenylase (Du *et al.*, 2016) or inducing endoribonucleolytic cleavage by the RNase P/MRP complex through the interaction of HRSP12 (Park *et al.*, 2019). Conversely, YTHDF1 enhances the translation of m6A mRNAs through its association with the translation initiation factors eiF3A/B and ribosomes (Wang *et al.*, 2015). YTHDF3 facilitates the translation of m6A mRNA through its binding of YTHDF1 (Li *et al.*, 2017a; Shi *et al.*, 2017) while facilitating m6A mRNA decay through binding of YTHDF2. In this model, YTHDF3 binds m6A mRNAs first, followed by the recruitment of YTHDF1 to promote translation, and lastly recruiting YTHDF2 to promote decay (Shi *et al.*, 2017). In support of this prevailing theory, YTHDF3 shares the most cognate mRNAs with both YTHDF1 and YTHDF2, whereas YTHDF1 and YTHDF2 share the least number of cognate mRNAs, excluding the mRNAs shared by all 3 YTHDFs (Shi *et al.*, 2017). Thus, YTHDF3 may be the true mechanism for selection and specificity of m6A mRNAs; however, the YTHDF paralogs share extremely high sequence similarity and bind m6A using a tryptophan (WWW) pocket (Li *et al.*, 2014; Patil *et al.*, 2018; Xu *et al.*, 2015; Zhu *et al.*, 2014), making it difficult to rely on their proposed cognate mRNA selectivity based on bioinformatics’ peak-calling methods that center on arbitrary thresholds (Patil *et al.*, 2018). A reanalysis of previously published PAR-CLIP experiments for YTHDF1-3 (Shi *et al.*, 2017; Wang *et al.*, 2014, 2015) (photoactivatable ribonucleoside-enhanced crosslinking and immunoprecipitation allows for nucleotide-level resolution identification of RNA-binding protein sites on target RNAs), and reassessment of siRNA knockdown of all 3 paralogs simultaneously, reported that these readers functioned only to promote the degradation of m6A mRNAs and were, thus, functionally redundant (Zaccara and Jaffrey, 2020). To support this redundant theory, in mouse embryonic stem cells, m6A mRNA half-life is unchanged after knockdown of any individual YTHDF, but simultaneous knockdown of all the YTHDFs results in a longer half-life of m6A mRNAs, suggesting compensation occurs in the event of single YTHDF knockdown (Lasman *et al.*, 2020). Therefore, the greatly conserved sequence similarity, the ability of the YTHDFs to bind each other, the tryptophan-binding mechanism, and the observed compensation support this notion of redundancy. If indeed the functions of all 3 YTHDFs are redundantly to promote degradation of m6A mRNAs, then it would be vital to seek and understand what the importance of such a redundancy is from an evolutionary aspect.

Here we highlight studies that have investigated any of these YTHDFs in a toxicological context to pave the way for how new studies should be conducted. Although in the cytoplasm, the paralogs display a generally diffuse expression, with evidence of YTHDF2 associating with processing bodies (p-bodies) in unstressed cells to promote m6A mRNA degradation (Du *et al.*, 2016; Ries *et al.*, 2019). Under specific stressed conditions, such as heat-shock or arsenite-induced oxidative stress, all paralogs have been observed to localize within stress granules (Ries *et al.*, 2019; Shi *et al.*, 2017; Wang *et al.*, 2014, 2015). This relocalization to stress granules depends upon the binding of polymethylated m6A mRNAs and their protein structure comprised of an ∼40-kDa, low complexity amino acid domain. Such domains are known to form fibrils, hydrogels, and liquid droplets during phase separation (Ries *et al.*, 2019). Note, YTHDF2’s localization to p-bodies also depends upon the binding of polymethylated m6A mRNA; however, when partitioned into stress granules by the phase transition mechanism, m6A mRNAs are not degraded. This supports the initial proposal that YTHDF1 could sequester mRNAs into stress granules. Then, after the resolution of stress, the mRNAs would be quickly translated because of YTHDF1’s interaction with translation initiation factors (Wang *et al.*, 2015). Nevertheless, phase separation of the paralogs demonstrates that the low complexity domain allows the polymethylated mRNA-bound YTHDFs to partition into various phase-separated structures under different cellular conditions, thereby promoting degradation of m6A mRNAs through p-body-localized enzymes while repressing m6A mRNA degradation when inside stress granules. This could be an enticing exploratory avenue of the paralogous readers from a toxicological perspective. P-bodies can be found in unstressed conditions (Ries *et al.*, 2019; Stoecklin and Kedersha, 2013), but they can also form under stressed conditions, and they can closely cluster with stress granules as in the case of arsenite treatment or the uncoupler of *oxidative* phosphorylation, FCCP (Stoecklin and Kedersha, 2013). Stress granules can also form in the presence of copper or high doses of hydrogen peroxide, both of which elicit oxidative stress (Emara *et al.*, 2012; Guarino *et al.*, 2019). Rotenone, a pesticide and mitochondrial complex I inhibitor known to generate high levels of oxidative stress in microglial cells (Sarkar *et al.*, 2017), also generates stress granules in the dopaminergic neurons of rats (Norazit *et al.*, 2010). These many perturbators of oxidative homeostasis in the formation of stress granules and p-bodies are generally studied in a transient context, however, novel perspectives highlight these cytoplasmic inclusions as formation models indicative of other pathological hallmarks such as α-synuclein and β-amyloid observed in PD and Alzheimer’s disease, respectively (Frydryskova *et al.*, 2020). Thus, investigating the YTHDF paralogs and their cognate m6A mRNAs in relation to stress granules and p-bodies may reveal vital mechanistic insights for chronic proteinopathies.

Most of the research on YTHDF paralogs beyond stress granules and p-bodies has predominantly been in cancer and virology. YTHDF2 has received much attention because of its initially proposed role in m6A mRNA decay. In a study of m6A methylation in hepatocellular carcinoma, hypoxic states were concomitant with reduced YTHDF2 levels, and this effect was predominantly exerted by HIF2α repression rather than HIF1α (Hou *et al.*, 2019). However, a previous study demonstrated YTHDF2 levels are restored in hypoxic states after siRNA knockdown of HIF1α in both the HEP3B and SMMC7721 hepatocellular carcinoma cell lines (Zhong *et al.*, 2019). Despite these differences, hypoxic states overall decrease the levels of YTHDF2, as significant reductions are achieved using cobalt chloride, a potent inducer of HIF1α and hypoxia (Zhong *et al.*, 2019). Nonetheless, YTHDF2 was deemed a tumor suppressor in both studies by promoting degradation of EGFR to suppress proliferation (Zhong *et al.*, 2019), and by promoting the degradation of IL-11 and SERPINE2, known secretory contributors for inflammatory invasion and metastasis (Hou *et al.*, 2019). In support, YTHDF2-depleted mouse macrophages develop enhanced inflammatory states post LPS treatment (Yu *et al.*, 2019), suggesting an anti-inflammatory role for YTHDF2, which targets MAP2K4 and MAP4K4 mRNAs, proteins known to activate p38, ERK, and NFκB signaling cascades. YTHDF1 has also been implicated in hypoxia and oxidative stress examined in lung cancer studies (Yu *et al.*, 2019; Shi *et al.*, 2019). YTHDF1 knockdown abolishes reactive oxygen species production generated by hydrogen peroxide in A549 adenocarcinoma. At the same time, NRF2 nuclear translocation was observed (Shi *et al.*, 2019). Additionally, the copper chelator, ammonium tetrathiomolybdate, increased YTHDF1 levels in A549 adenocarcinoma at low concentrations, suggesting YTHDF1 could promote lung cancer growth (Li *et al.*, 2020). Lastly, YTHDF3 gets downregulated in HTR8/SVneo trophoblast cells during hypoxia induction (Zheng *et al.*, 2020) and by cobalt chloride in C2C12 mouse myoblasts (Chen *et al.*, 2019c). All the aforementioned data suggest the cytoplasmic YTHDF paralogs have significant functions under various toxicological stressors and commonly under hypoxic conditions prevalent in many toxicopathologies.

## FUTURE DIRECTIONS

Herein, we have discussed the principal tiers of m6A mRNA regulation from a toxicological perspective, beginning with the co-transcriptional deposition of methyl groups, the potential erasure of methyl modifications, and ending with their decreased stability and decay. Other domains of m6A regulation do exist such as those pertaining to splicing and mRNA export. In these processes, YTHDC1 can regulate pre-mRNA splicing via recruitment of splicing factors, and its knockdown can prevent m6A mRNA export (Kasowitz *et al.*, 2018; Roundtree *et al.*, 2017; Roundtree and He, 2016; Xiao *et al.*, 2016). Different heterogeneous nuclear ribonucleoproteins can also affect alternative splicing events and the processing of precursor or primary microRNAs (Alarcon *et al.*, 2015; Liu and Pan, 2016). Furthermore, YTHDC2 is the largest member of the YTH-domain family of proteins that contains a helicase domain. It has been observed to participate in both *m6A* mRNA translation and acceleration of decay through interactions with XRN1 exonuclease (Hsu *et al.*, 2017; Kretschmer *et al.*, 2018; Mao *et al.*, 2019). The cellular circumstances and pathways discussed in the 3 tiers of regulation clearly show the importance of the m6A epitranscriptomics. Notably, we highlight the common involvement of all 3 regulatory tiers in the cellular context of hypoxia. Different stressors were exemplified in generating hypoxic states whose effects ranged from decreasing to increasing global m6A methylation. Because these global m6A signatures depended on the duration and exposure of each specific toxicant/treatment, it remains difficult to predict the effects of insults on global m6A signatures, especially as they can also vary across toxicological and exposure paradigms. To provide an overall templated guide for future research, we compiled a table of global m6A methylation changes observed under different toxicant/treatment paradigms, including the loss-of-function changes associated with the different m6A proteins (Table 1). We caution that changes in global m6A methylation are not necessary in the cases of differential regulation amongst the writers, erasers, and readers. Pertaining to methodologies for assessing m6A levels, we do advocate for the most stringent and sensitive methods consisting of LC-MS/MS or crosslinked sequencing when applicable.

**Table 1.** Global m6A Methylation Changes in Different Experimental Paradigms

Toxicants/Treatments	Model System	Global m6A	Reference (PMCID)
6-Hydroxydopamine	PC-12 and *Rattus norvegicus* Striatum	Decrease	30835997
Aflatoxin B1	*Mus musculus* liver	Increase	32294948
Ammonium tetrathiomolybdate	A549	Increase	32195181
Arsenite (high concentration)	HaCaT	Decrease	30654086
Arsenite (long-term exposure)	HaCaT, A549	Decrease	31931413, 31146095
Arsenite (low concentration)	HaCaT	Increase	30654086
Arsenite (short-term exposure)	NIH3T3	Insignificant	31292544
Bisphenol A	A549, Danio rerio Larvae (120-hpf)	Decrease	31146095, 32143076
Chidamide	HCC827, H661, A549, H1650	Decrease	32792859
Colistin	mRTEC	Decrease	31156435
Di-(2-ethylhexyl) phthalate	*Rattus norvegicus* prepubertal testes	Increase	31923814
Fluorene-9-bisphenol	*Danio rerio* Larvae (120-hpf)	Decrease	32143076
Heat shock	Mouse embryonic stem cells	Insignificant	31292544
Heat shock	MEF, MEF	Increase (5′ UTR)	26593424, 26458103
Hypoxia	HEK-293T	Increase	28611253
Lipopolysaccharide	Human dental pulp cells, pTHP-1	Increase	29502358, 31772500
Meclofenamic acid	HeLa	Increase	25452335
Mono-(2-ethylhexyl)phthalate	Raw 264.7	Decrease	31875672
N‐acetylpaminophenol	*Mus musculus* liver	Increase	31197931
Particulate matter (1648a)	A549	Decrease	31146095
Particulate matter_2.5_	*Mus musculus* lungs	Increase	30731271
siALKBH5	HeLa	Increase	23177736
siFTO	HeLa and 293FT, HEK293T	Increase (5′ UTR)	22002720, 28002401
siMETTL14	HeLa and 293FT	Decrease	24316715
siMETTL3	HeLa and 293FT	Decrease	24316715
siWTAP	HeLa and 293FT	Decrease	24316715
siYTHDF1	HeLa, HeLa	Insignificant	26046440, 28106072
siYTHDF1/2/3	HeLa	Increase	28106072
siYTHDF2	HeLa, HeLa	Increase	24284625, 28106072
siYTHDF3	HeLa	Insignificant	28106072
Triclosan	*Danio rerio* larvae (120 hpf)	Decrease	32143076
Ultraviolet C irradiation	U2OS	Increase	28297716
Vinclozolin	A549	Decrease	31146095

Published works are listed that investigated global m6A changes in toxicological paradigms. Additionally, small-interfering RNAs of the writers, erasers, and readers have been placed for comparison. Although the majority of studies utilized LC-MS/MS to quantify global m6A methylation changes, other studies utilized either m6A fluorometric methods or m6A-sequencing with bioinformatic processing. Please see references for these method details.

**Figure 2.**
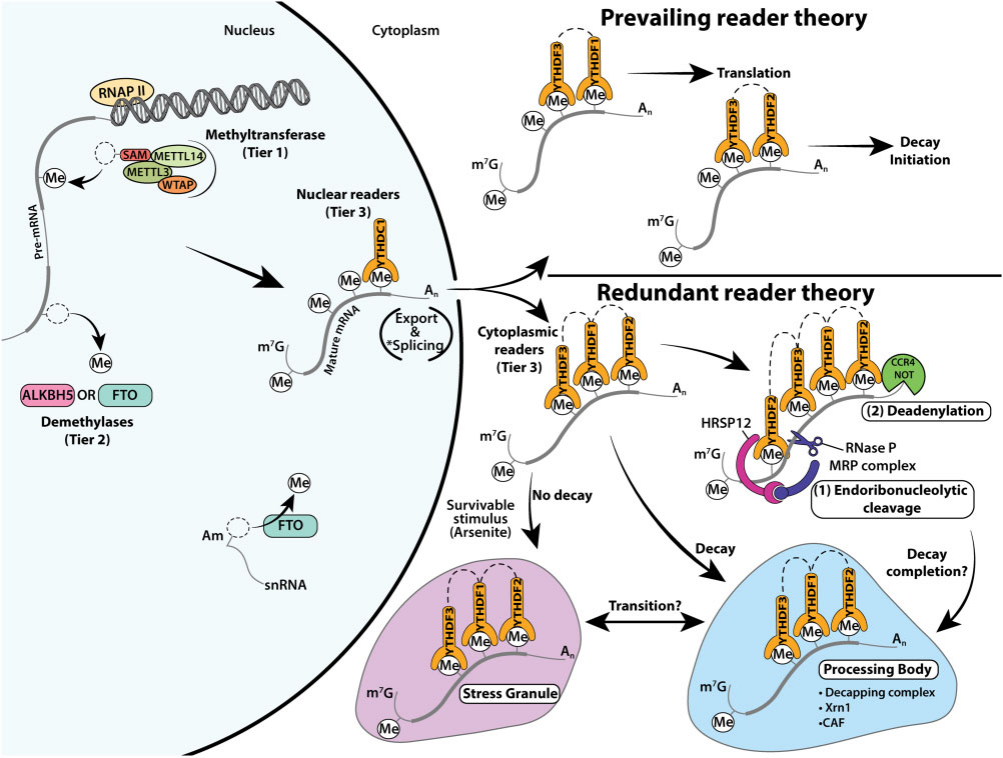
Synthesis of m6A and tiers of its regulation contrasting the prevailing reader theory and the redundant reader theory. During transcription, the methyltransferase writer complex takes methyl groups from SAM and deposits them onto DRACH consensus sequences. Although in the nucleus, methylated mRNAs can be encountered by erasers such as ALKBH5 and FTO (targets small nuclear RNAs and m6Am), or by reader YTHDC1 that can regulate splicing (*occurs on pre-mRNAs) and export from the nucleus. Under the prevailing reader theory, the polymethylated mRNAs are bound by YTHDF3 and YTHDF1 for translation, whereas YTHDF3 and YTHDF2 target them for degradation. Prevailing evidence suggests these polymethylated mRNAs are processed rapidly and sequentially, first being bound for translation and then followed by decay. Under the redundant reader theory, the YTHDFs bind their cognate mRNAs to only and specifically initiate degradation through either the (1) endoribonucleolytic cleavage pathway, via interaction with HRSP12 (recognizes GGUUC sequence) and RNase P/MRP complex, or the (2) deadenylation pathway via interaction with CCR4-NOT. YTHDF binding of polymethylated mRNAs allows for phase separation and partitioning through the binding of their low-complexity domains (dash connections), principally guided to p-bodies. Evidence suggests degradation occurs within p-bodies or may instead be the location of the final stages of mRNA degradation after the initial processing through either endoribonucleolytic cleavage or deadenylation. Upon survivable stressors such as arsenite, YTHDFs bind polymethylated mRNAs and can partition them into stress granules rather than p-bodies, resulting in no degradation of these bound cognate mRNAs. Lastly, how and when transitioning between p-bodies and stress granules occurs and what effects this confers upon the entangled m6A mRNAs remains to be further evaluated.

Our review also addresses the controversial demethylase activities of FTO, and to a larger extent, the functional theories of the cytoplasmic YTHDF readers. Though FTO’s *k*_cat_ is measurably greater for m6Am than for m6A, the nuclear domain is a small and relatively constrained space where molecular proximity strongly influences molecular interactions. It is conceivable that FTO is specific toward demethylating m6Am small nuclear RNAs, but because of molecular proximity, FTO can indeed execute demethylation of m6A mRNAs during encounters despite the contradictory evidence. Lastly, whether the YTHDF paralogs confer different reading functions, or if they all functionally converge to promote the decay of m6A mRNAs is an intriguing and vital biological question. We provide a schematic (Figure 2) on m6A biology that reflects the prevailing and redundant theories of YTHDF reading. For all future studies concerning the readers, we caution researchers to assess the expression levels of all YTHDF proteins in their experimental paradigms to better explicate the behaviors of the readers. Nevertheless, we encourage researchers to embrace this new area of epitranscriptomics that will provide new insights into the etiopathogenesis of environmentally linked chronic diseases.
